# *Vibrio gazogenes-*dependent disruption of aflatoxin biosynthesis in *Aspergillus flavus*: the connection with endosomal uptake and hyphal morphogenesis

**DOI:** 10.3389/fmicb.2023.1208961

**Published:** 2023-09-08

**Authors:** Rubaiya Jesmin, Jeffrey W. Cary, Matthew D. Lebar, Rajtilak Majumdar, Phani M. Gummadidala, Travis Dias, Savannah Chandler, Paramita Basu, Alan W. Decho, Nancy P. Keller, Anindya Chanda

**Affiliations:** ^1^Mycologics LLC, Frederick, MD, United States; ^2^Food and Feed Safety Research Unit, USDA/ARS, Southern Regional Research Center, New Orleans, LA, United States; ^3^Northwest Irrigation and Soils Research, United States Department of Agriculture, Kimberly, ID, United States; ^4^University of North Carolina School of Medicine, Chapell Hill, NC, United States; ^5^University of South Carolina School of Medicine, Greenville, NC, United States; ^6^Department of Environmental Health Sciences, University of South Carolina, Columbia, SC, United States; ^7^New York College of Podiatric Medicine, New York, NY, United States; ^8^Department of Medical Microbiology and Immunology, Department of Plant Pathology, University of Wisconsin-Madison, Madison, WI, United States

**Keywords:** aflatoxin, mycotoxin, *Aspergillus flavus*, biocontrol, endosomes

## Abstract

Aflatoxins, a family of fungal secondary metabolites, are toxic and carcinogenic compounds that pose an enormous threat to global food safety and agricultural sustainability. Specifically agricultural products in African, Southeast Asian and hot and humid regions of American countries suffer most damage from aflatoxin producing molds due to the ideal climate conditions promoting their growth. Our recent studies suggest that *Vibrio gazogenes* (Vg), an estuarine bacterium non-pathogenic to plants and humans, can significantly inhibit aflatoxin biosynthesis in the producers. In this study, we investigated the mechanism underlying Vg-dependent aflatoxin inhibition using the prominent aflatoxin producer, *Aspergillus flavus*. We show that aflatoxin inhibition upon Vg treatment was associated with fungal uptake of Vg-prodigiosin, a red pigment, which was consistently visible inside fungal hyphae during treatment. The association of prodigiosin with aflatoxin inhibition was further evident as *Serratia marcescens*, another prodigiosin producer, significantly inhibited aflatoxin, while non-producers like *Escherichia coli, Staphylococcus aureus*, *Vibrio harveyi*, and *Vibrio fischeri* did not. Also, pure prodigiosin significantly inhibited aflatoxin biosynthesis. Endocytosis inhibitors, filipin and natamycin, reduced the Vg-prodigiosin uptake by the fungus leading to a significant increase in aflatoxin production, suggesting that uptake is endocytosis-dependent. The Vg treatment also reduced hyphal fusion (>98% inhibition) and branching, which are both endosome-dependent processes. Our results, therefore, collectively support our theory that Vg-associated aflatoxin inhibition is mediated by an endocytosis-dependent uptake of Vg-prodigiosin, which possibly leads to a disruption of normal endosomal functions.

## 1. Introduction

Aflatoxin and many other fungal toxic secondary metabolites (mycotoxins) contaminate various foods and crops worldwide, posing a significant threat to production of agricultural products, food safety, public health, and the global economy (Kos et al., 2023). Specifically aflatoxins are known to contaminate a wide range of crops including grains, tree nuts, oilseeds, and spices, in hot and humid regions of the world where environmental factors (such temperature, relative humidity, rainfall, and soil types) and pre- and post-harvest management practices and conditions promote fungal growth and mycotoxin synthesis (Hell and Mutegi, 2011). The subtropical and tropical regions of Africa and south east Asia have suffered the most aflatoxin contamination problems in crops and this has also been exacerbated by inadequate drying during long-term storage, poor aeration, temperature control and insect infestations (Misihairabgwi et al., 2019). However, with increasing global warming, aflatoxin is now becoming an imminent threat to agricultural sustainability and human and animal health in previously unaffected regions in Europe and North America as well (Adeyeye, 2020; Fisher et al., 2020; Meyer et al., 2020; Ráduly et al., 2020; Sharma and Bhandari, 2020). For example, aflatoxin, a mycotoxin produced primarily by *Aspergillus flavus*, is highly toxic and a potent naturally occurring carcinogen (Wild and Gong, 2010; Coppock et al., 2018), and contamination levels are predicted to increase due to impending climate change (Battilani et al., 2016). Currently, estimated US losses range from $52.1 million to $1.68 billion annually for aflatoxin alone (Mitchell et al., 2016). Unlike the United States and several developed nations within the European Union, the implementation of the stringent quality control methods for lowering the dietary exposure to mycotoxins is not always feasible. Hence mycotoxin contamination in food and feed pose a major health threat in many underdeveloped and developing countries worldwide (Imade et al., 2021; Marimon Sibaja et al., 2022; Bhardwaj et al., 2023; Sun et al., 2023). Despite years of mitigation efforts, over 5 million people worldwide are at risk of dietary exposure to just aflatoxin (Gummadidala et al., 2019; Eskola et al., 2020).

Fungicidal chemicals have conventionally been used for inhibiting fungal pathogens in agriculture. However, there is a growing interest in newer generation farmers to reduce their dependence on these chemicals due to the concerns of residual effects and toxicity that affect the environment and human health. Also, consumers commonly perceive pesticide chemicals as a threat, and there are new laws in place that either ban or restrict their use by imposing lower maximum residue limits. To reduce pesticide overuse, the concept of integrated pest management (IPM) has emerged, which is the combinatorial use of safe plant disease control strategies to obtain the best crop yield and profit (Kagot et al., 2019). According to IPM principles, a combined application of biological control agents with fungicides will reduce the fungicide dose or the frequency of application and improve disease control (Palumbo et al., 2006; Khalid et al., 2018; Spraker et al., 2018). It will also reduce the selection pressure on pathogens and thereby the chances of resistance development. To address mitigation of mycotoxin contamination in crops, the use of microflora and their metabolites as biocontrol agents against *A. flavus* and other mycotoxigenic fungi are becoming increasingly popular (Palumbo et al., 2006; Khalid et al., 2018; Spraker et al., 2018; Kagot et al., 2019): examples include studies with atoxigenic *A. flavus* (Agbetiameh et al., 2019; Ezekiel et al., 2019; Ortega-Beltran et al., 2019) *Bacillus* spp. (Chalivendra et al., 2018; Guo et al., 2019; Liu et al., 2019), *Lactobacillus* spp. (Ahlberg et al., 2017; Guimaraes et al., 2018; Gonda et al., 2019), *Pseudomonas* spp. (Yang et al., 2017; Kagot et al., 2019; Wang et al., 2019) and *Ralstonia* spp. (Spraker et al., 2014, 2016). The current study was initiated in response to a call from the National Corn Growers Association (NCGA) of the United States to identify effective biocontrol agents having the potential to combat the persistent profit robbing aflatoxin issues that the U.S. corn growers identified as an urgent problem (NCGA, 2021) despite having access to the potential alternative solutions.

Our recently published findings suggest that the marine Gram-negative bacterium, *Vibrio gazogenes* (denoted here as Vg), is capable of inhibiting aflatoxin biosynthesis in the aflatoxin producers: >2-fold reduction in *A. parasiticus* (Gummadidala et al., 2016) and > 95% reduction in *A. flavus* (Kandel et al., 2022). However, the mechanism of such inhibition has not been explored thus far and is critical to understand before Vg can be used for field applications to control aflatoxin contamination. Hence, in this study, we sought to examine the mechanism of Vg-dependent aflatoxin inhibition in *A. flavus*. The specific aims of this study were (Misihairabgwi et al., 2019) to examine the effects of intact Vg cells and its secondary metabolite, prodigiosin on aflatoxin production; and (De Ruyck et al., 2015) to understand if the effects of the treatments were associated with the hyphal endocytic uptake of either Vg or its secondary metabolite, prodigiosin.

## 2. Materials and methods

### 2.1. Strains and culture conditions

*Vibrio gazogenes*, ATCC 43942, was used in this study and was grown in Difco Marine Broth 2216 (BD Biosciences, Sparks, MD, United States) at 28°C in a shaking incubator (190 rpm) in the dark for 24 h before harvesting the cells for interaction experiments. This condition rendered ~1.6e^7^ Vg cells after harvest that we used for all treatments in this study (unless other concentrations are mentioned under the specific experiments). Other bacterial strains used included *Serratia marcescens* (ATCC 39006), *Escherichia coli* (ATCC 25922), *Staphylococcus aureus* (ATCC 25923), *Vibrio harveyi* (ATCC 14126), and *Vibrio fischeri* (ATCC 7744). Vibrio strains were grown in Marine Broth at 28°C in the dark in a shaking incubator (190 rpm) for 24 h, while the other strains were grown in Nutrient Broth in the dark at 37°C at 250 rpm for 12 h before harvesting. Under these conditions, all bacterial strains produced equal concentrations of cells as determined for Vg (~1.6e^7^ cells total). Fungal strains used in this study were stored as glycerol stocks at −80°C until they were needed. For the fungal experiments, the *A. flavus* wild-type strains NRRL 3357 or SRRC1529 (also known as AF70) strain were used. YES (6% sucrose, 2% yeast extract, pH 5.8) medium was used for experiments with liquid culture, and potato dextrose agar (PDA) was used for experiments with solid culture. All fungal strains were grown on glucose minimal medium (GMM) for spore production at 29°C. In some experiments using the *pyrG* auxotrophic strain, TJES 19.1, 0.56 g/L uracil, and 1.26 g/L uridine were supplemented, and for the *argB* auxotrophic strain, TJES 20.1, 1 g/L arginine was supplemented in the growth medium. In some cases, all three supplements were added, as described below. Heterokaryon analysis was assessed on GMM + 0.25% Triton X-100 medium.

### 2.2. *Aspergillus flavus* treatments with Vg and other bacteria

Unless otherwise specified, all Vg treatments consisted of 24 h marine broth cultures of Vg that were pelleted by centrifugation at 4000 *g* for 15 min at room temperature (25°C), then resuspended in 1 mL YES media (~1.6e^7^ cells). This suspension was added to 100 mL YES growth medium inoculated with 10^7^ spores/mL of *A. flavus* and incubated in the dark at 29°C and 150 rpm for 40 h. For generating heat-inactivated and non-viable Vg and other bacteria (*E. coli*, *S. aureus*, *V. harveyi*, *V. fischeri* cells, and *S. marcescens*), the cultures were resuspended in 1 mL YES broth and incubated at 100°C for 10 min. Non-viability was confirmed by plating the heat-treated cells on bacterial growth medium (Marine agar at 29°C in the dark for Vibrio strains and Nutrient Agar at 37°C in the dark for other bacterial strains) and ensuring that no growth occurred over 24 h. Vg treatment of *A. flavus* on agar plates was conducted by center-point inoculating 10 μL (total of ~10^3^ fungal spores) on the plate previously spread with ~1.6e^7^ cells/mL of the bacteria. Studies on viable corn seed (inbred maize line Va35) were conducted by pooling three sets of 10 surface-sterilized seeds per experimental group and treating as follows: (i) uninoculated and untreated, (ii) *A. flavus* inoculated but untreated, and (iii) inoculated and treated with Vg. For the treatment group, 1.6 × 10^7^ Vg cells suspended in 1 mL YES media were applied to the seeds and semi-dried prior to the fungal spore inoculation. All groups were incubated at 30°C in the dark for 5 days prior to aflatoxin analysis.

### 2.3. Fungal imaging experiments

For light imaging experiments, *A. flavus* spores (~10^2^ spores) were inoculated in 20 μL of YES growth medium in a glass-bottom dish with or without Vg added to the growth medium. A widefield microscope (Finite Optical System, Fischer Scientific) was used for bright-field microscopy. *A. flavus* colonies growing on PDA with or without Vg treatment were visualized by stereomicroscopy (EZ4 W, Leica microsystems). Mycelial pellets harvested at 40 h post-inoculation were used for transmission electron microscopy (TEM) studies. For TEM, the pellets were fixed using 3% glutaraldehyde and 2% osmium tetroxide, dehydrated in an ethyl alcohol series, and made into resin blocks. The resin blocks were trimmed and cross-sectioned into approx. 80 nm thick sections using a diamond knife (Micro Star Technology Inc., Huntsville, Texas) on a Sorvall Porter-Blum MT2-B Ultra-Microtome. Sections were loaded onto copper grids and later stained with lead citrate and 5% uranyl acetate. The stained grids were then imaged using a Hitachi H8000 TEM with pressure maintained at 10^−7^ Torr and an accelerating voltage maintained at 200 kV.

### 2.4. Growth of *Aspergillus flavus* on prodigiosin-supplemented growth medium

A total of 250 mL of YES agar was divided into five 50 mL aliquots and autoclaved. Pure prodigiosin (Cayman Chemical Co., Ann Arbor, MI) was added from 1 mg/mL stock to every 50 mL of tempered YES agar medium to yield final concentrations of 0, 0.5, 1.0, 2.0, and 4.0 μg/mL prodigiosin. Ten milliliter aliquots of the prodigiosin supplemented YES agar medium was added to five 60 × 15 mm Petri plates for each concentration of prodigiosin used. The dishes were center-point inoculated with 2 μL of a 1e^7^ solution of AF70 spores and placed at 30°C for 3 days in the dark. After the incubation period, colony diameter was measured and then agar was excised and extracted for determination of aflatoxin levels.

### 2.5. Hyphal fusion assay

The influence of Vg treatment on the hyphal fusion process was investigated using an established assay described previously (Zhao et al., 2017). Briefly, equal concentrations of the *A. flavus pyrG* auxotrophic strain (TJES 19.1) and *argB* auxotrophic strain (TJES 20.1) were mixed and spotted onto GMM amended with arginine (1 g/L) and uracil (5 mM)/uridine (5 mM) with or without 1.6e^7^ Vg. After incubation at 29°C for 5 days, the newly formed conidia were collected in Phosphate Buffered Saline, and 10^5^ conidia were spread onto the surface of GMM + 0.25% Triton X-100 agar plates (the 0.25% Triton X-100 restricts colony diameter to help with precise colony counts). Heterokaryotic colonies were counted after incubation for 3 days at 29°C. A cross of the hyphal anastomosis mutant, Δ*hamI* -TXZ9.16 with TJES 20.1, was used as a negative control.

### 2.6. Colony diameter measurements and hyphal branching comparisons

Colony diameters were measured by assessing the mean distance from the outer edge of the growing colony to the center of the colony following growth on PDA for 5 days at 30°C in the dark. For comparing the extent of hyphal branching between the Vg-treated and untreated colonies, the colonies grown on PDA were observed under a stereomicroscope (EZ 4w, Leica Microsystems) and a scoring method used to quantify the visible differences in hyphal branching under the microscope. Twenty investigators were asked to assess a control (untreated) *A. flavus* colony and a Vg-treated colony. In a blinded fashion, they were asked to assign a score of 0–10, with 0 representing no branching and 10 representing the extent of branching observed in control.

### 2.7. Treatment with endocytosis inhibitors

Two polyene endocytosis inhibitors, filipin and natamycin (Sigma Chem. Co, St. Louis), were used to study their effect on Vg-PG uptake and aflatoxin production. Stock solutions (1 mmol/L) of the inhibitors were made using DMSO. We used filipin at 3 μmol/L and natamycin at 6 μmol/L for treatments. These doses corresponded to the minimal inhibitory concentrations (MICs) previously observed for the disruption of *Aspergillus* endosomal development (Van Leeuwen et al., 2009).

### 2.8. Color scoring assay for comparison of Vg-prodigiosin uptake

Since mycelial uptake of Vg-PG correlates with the transition from normally non-pigmented fungal hyphae to a reddish color upon complete uptake at 40 h, the effect of the inhibitors on Vg-PG uptake was assessed by visual monitoring of the mycelia and by comparing its color to the control mycelia. A less intense red color corresponded to less uptake. To determine whether the inhibition of Vg-prodigiosin uptake was statistically significant, we developed a blinded visual scoring method to conduct a quantitative comparison of Vg-prodigiosin uptake in the presence and absence of endocytosis inhibitors. A total of 20 separate examiners, who had no prior knowledge of the samples, were asked to provide a color intensity score to 40 h mycelial pellets representing untreated control (displaying noninhibited Vg-prodigiosin uptake) and the natamycin and filipin-treated pellets (displaying inhibited Vg-prodigiosin uptake). Examiners were asked to provide a color score on a scale of 0 to 10, where 0 indicated no red color observed in mycelial pellets, as seen in 40 h untreated pellets (negative control), and 10 indicated entirely red mycelial pellets, corresponding to uninhibited Vg-prodigiosin uptake (positive control). The choice of 40 h old pellets for color scoring was based on the rationale that mycelia show the highest color intensity (corresponding to an almost complete Vg-PG uptake) at this time point under our culture conditions. A higher red color score, therefore, represented higher Vg-PG uptake.

### 2.9. Statistical analysis

All statistical tests were performed using GraphPad Prism Software (GraphPad, La Jolla, CA, USA). Statistical analysis was carried out using one-way ANOVA followed by Tukey’s multiple comparison test. Statistical significance of difference was set at *p* < 0.05.

### 2.10. Fungal biomass measurements

Fungal dry weight was measured as described previously (Kenne et al., 2018) by collecting mycelia from the growth medium and then drying the mycelial mass in an oven at 80°C for 6 h. Weight difference before and after drying was recorded as the dry weight.

### 2.11. Aflatoxin extraction and quantification

Aflatoxin was extracted from liquid and solid cultures using a standard chloroform-methanol extraction protocol described previously (Kenne et al., 2018). In addition, as described previously (Gummadidala et al., 2019), aflatoxin was quantified in the extracts using ELISA (with limit of detection at 2.5 ppb) and HPLC (with limit of detection at 0.1 ppb).

## 3. Results

### 3.1. Effect of Vg on aflatoxin production

**Figure 1:**
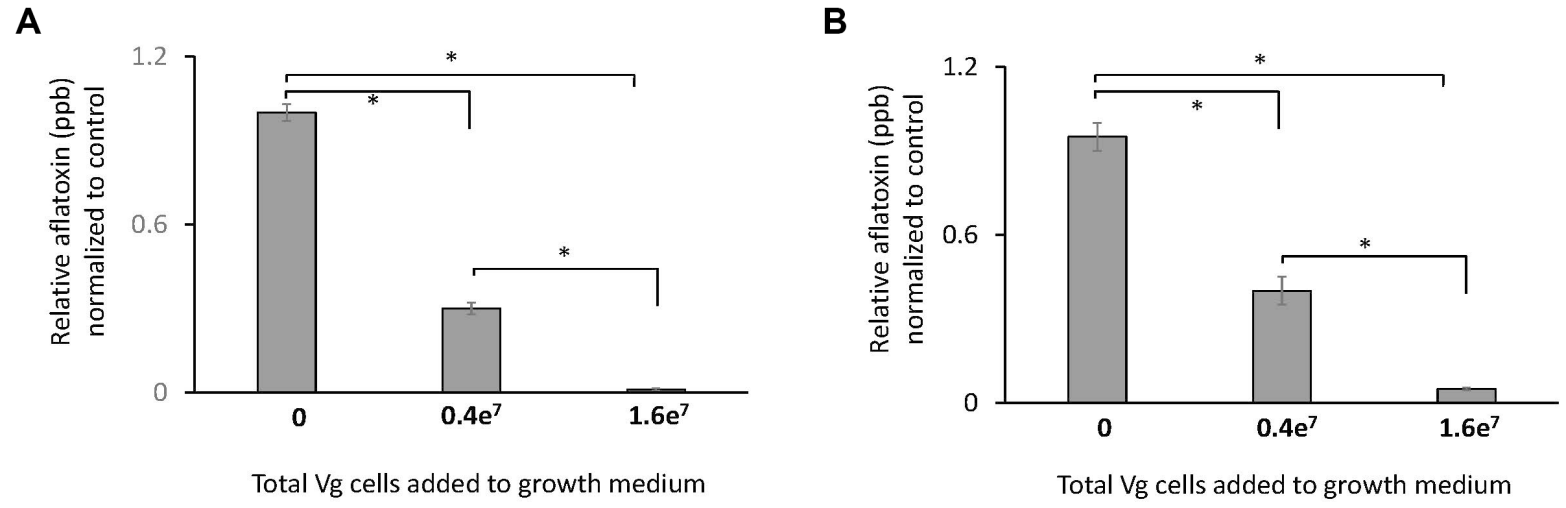
Effect of Vg on aflatoxin production. Aflatoxin produced per dry weight by *A. flavus* NRRL 3357 in **(A)** liquid yeast extract sucrose (YES) growth media and **(B)** yeast extract sucrose (YES) agar growth media were compared in the presence and absence of Vg. Two doses of bacterial cells, 0.4e^7^ total cells (4e^4^ cells/mL medium) and 1.6e^7^ (1.6e^5^ cells/mL medium), were used for the study. Aflatoxin was quantified by ELISA assay after 40 h. The values in the y-axis show the relative aflatoxin levels normalized to the untreated controls. Error bars, SEM, from triplicate experiments. Statistical significance of two-tailed *p*-values determined using one-way ANOVA for n = 3. (*statistically significant difference with *p* < 0.05).

Two doses of Vg were used to treat *A. flavus* growing in liquid and solid growth media. Results showed that the administration of 1.6e^7^ bacterial cells resulted in an almost complete inhibition (>99%) of aflatoxin production both in liquid (Figure 1A) and solid (Figure 1B) growth media. The lower dose (0.4e^7^ cells) resulted in less inhibition (~50% reduction) of aflatoxin production. Due to the more significant observed reduction in aflatoxin production, 1.6e^7^ Vg cells were used for all subsequent experiments. No significant effects on total fungal biomass as measured by dry weight was observed upon Vg treatments (data not shown). Studies were also performed to determine if viable Vg was essential for Vg-dependent aflatoxin reduction. As shown in Supplementary Figure S1, treatment using heat-treated, non-viable Vg resulted in a similar reduction (>95%) of aflatoxin production as treatment with viable Vg. In a separate experiment, Vg, when applied to *A. flavus* infected corn seeds, resulted in a similar reduction (>90%) in aflatoxin (Supplementary Figure S2), which supports the idea that Vg could restrict aflatoxin contamination in crops in a manner similar to our batch experiments using artificial media.

### 3.2. Mycelial uptake of Vg-prodigiosin

**Figure 2:**
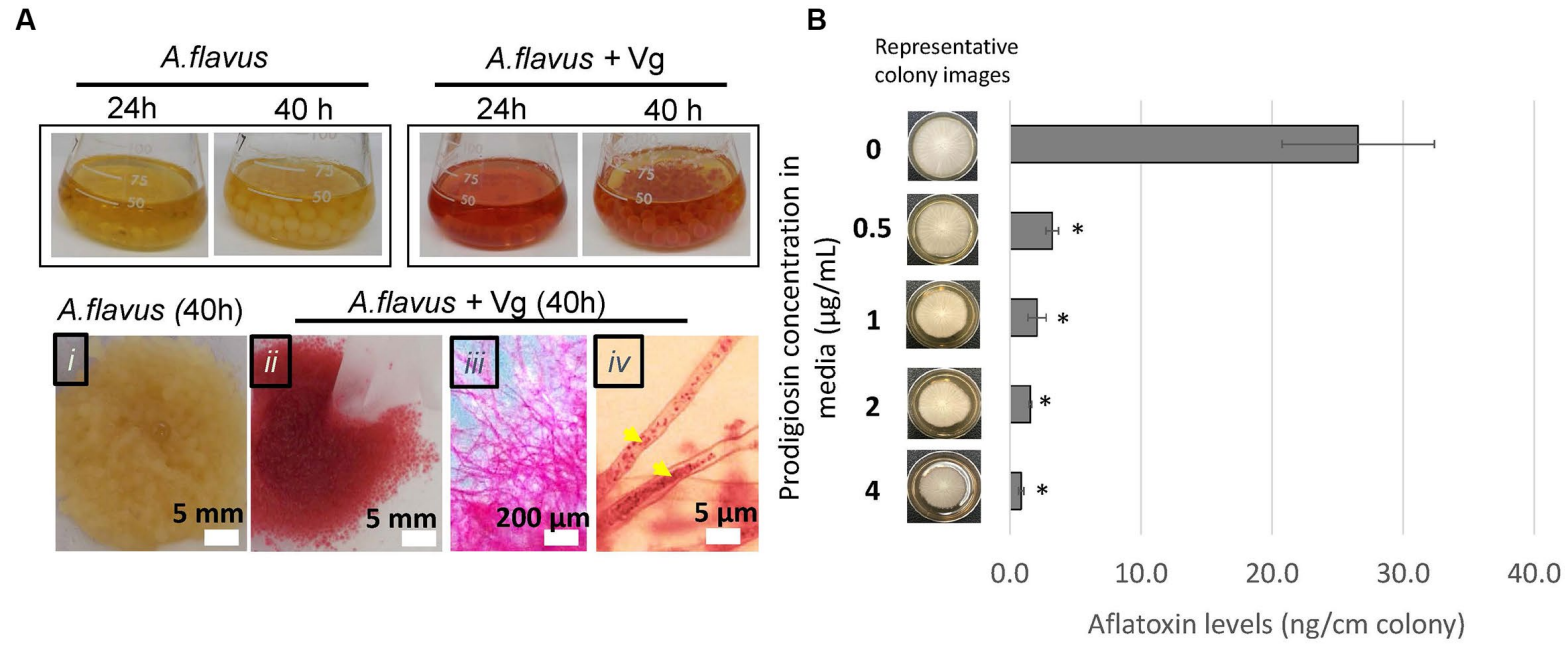
Vg prodigiosin influence on aflatoxin production. **(A)** Vg-prodigiosin uptake by *A. flavus*. *Upper panel*. Photographs of flasks with 24 h and 40 h fungal growth cultures with and without Vg treatment. *Upper panel*, photographs of 40 h harvested fungal cultures: (i) untreated (control) fungal culture, (ii‑iv) Vg-treated fungal cultures, where (iii) shows a magnified image of a mycelial section, and (iv) shows a magnified image of hyphae with internalized Vg prodigiosin (yellow arrows). **(B)** Influence of pure prodigiosin on aflatoxin production. *A. flavus* center-inoculated on YES agar supplemented with various concentrations of PG was allowed to grow for three days at 30°C in the dark before the representative images of colonies were taken, and aflatoxin was quantified in the cultures. Error bars represent SEM, and the statistical significance of two-tailed *p*-values was determined using one-way ANOVA for *n* = 5. (*p* < 0.05), *statistically significant difference compared to untreated colonies.

During Vg treatments, we consistently observed that by the 40-h time-point, the fungal mycelia turned red (a color similar to Vg-prodigiosin that we denote here as Vg-PG; see Figure 2A). Bright-field microscopy also indicated the presence of Vg-PG at discrete locations along the hyphae (see hyphal image after Vg treatment in Figure 2A), suggesting a hyphal uptake of Vg or a Vg-PG enriched fraction. TEM images of hyphae of Vg-treated and untreated *A. flavus* cultures showed no trace of intact rod-shaped Vibrio cells inside Vg- treated hyphae (Supplementary Figure S3), confirming that intact uptake of Vg did not occur. During the Vg treatment we also observed a steady decrease of Vg as reflected by the OD600 readings of the treatment broth until it reached non-detectable levels by 40 h (Supplementary Figure S4), suggesting a possibility of bacteria lysis in the growth medium during co-culture, that could explain why no signs of intact Vg cells were observed in TEM images.

### 3.3. Influence of prodigiosin on aflatoxin inhibition

Next, we proceeded to examine the influence of prodigiosin on aflatoxin production by the fungus. First, we treated *A. flavus* with different bacteria (including Vg) to compare the effects of the treatments on aflatoxin production. The non-Vg bacteria included *V. harveyi* and *V. fischeri* (two *Vibrio* spp. that do not produce prodigiosin), *S. marcescens* (a non-Vibrio prodigiosin producer), *E. coli* (as a representative Gram-negative bacterium) and *S. aureus* (a representative Gram-positive bacterium). As shown in Supplementary Figure S5, significant aflatoxin reduction compared to untreated control was observed only upon treatment with the prodigiosin producers, Vg and *S. marcescens*. To determine whether prodigiosin was the reason for the specificity of aflatoxin inhibition we then tested the ability of pure prodigiosin to inhibit aflatoxin production. As shown in Figure 2B, aflatoxin was significantly inhibited at all levels of prodigiosin with a ~ 88–97% decrease corresponding to 0.5–4.0 ug/mL pure prodigiosin. In addition, some inhibition of fungal growth as measured by colony diameter was observed at all levels but not enough to account for the observed decreases in aflatoxin production. Collectively, the data imply that prodigiosin is one critical bioactive component linked with Vg-dependent aflatoxin inhibition.

### 3.4. Effect of vg treatment on hyphal fusion and branching

**Figure 3:**
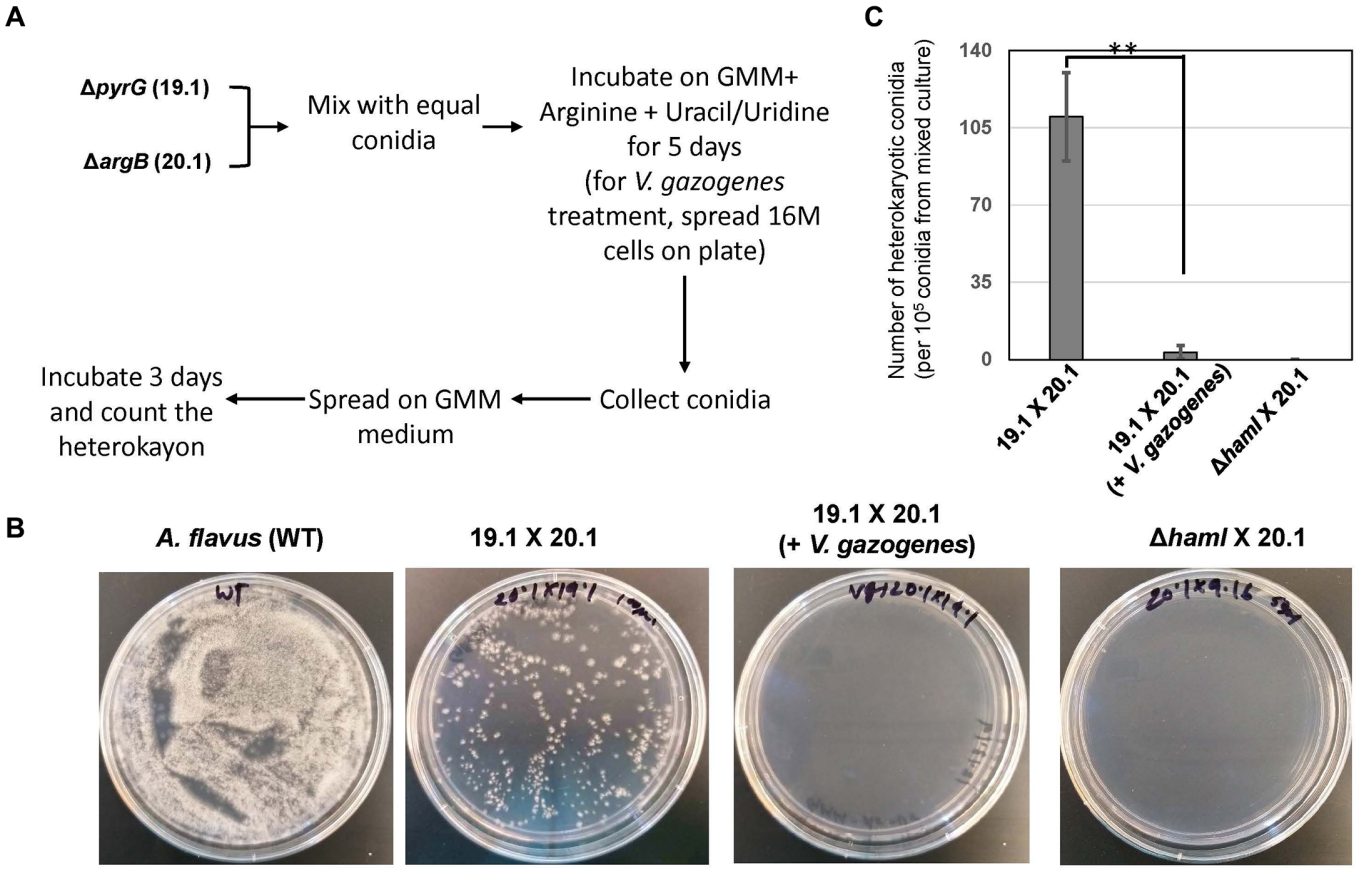
Influence of *V. gazogenes* treatment on hyphal fusion. **(A)** Outline of the method to measure hyphal fusion based on the method described by Zhao et al. **(B)** Photographs of heterokaryon formation or lack of formation in crosses. Plates of mixed cultures were spread and incubated at 29°C for 3 days. **(C)** The number of heterokaryotic colonies formed for each cross. **p < 0.001. *ΔhamI* (TX29. 16) was uracil/undine auxotrophic. 19.1 (TJES19.1), uracil/undine auxotrophic. 201 (TJES20 1), arginine auxotrophic.

Based on the observations of Vg-PG localized at discrete locations within fungal hyphae during Vg treatment (in Figure 2A), further investigation was conducted to test if Vg treatment influenced hyphal fusion. Hyphal fusion is a fundamental biological process in filamentous fungi coordinated by fungal endocytic machinery and endosomal transport (Riquelme et al., 2018; Fischer and Glass, 2019). Hence we reasoned that if Vg-PG internalized in endosomes and thereby disrupted endosomal functions, hyphal fusion would be disrupted in a Vg-dependent manner. Results from the hyphal fusion assay (Figure 3) indicated that the process of heterokaryon formation from crosses between TJES 19.1 and TJES 20.1 was almost completely inhibited (by >95%) in the presence of Vg. This observation was similar to the negative control (crosses between the hyphal anastomosis mutant, *ΔhamI*, and TJES 20.1). In addition, Vg treatment’s influence on hyphal branching within the colony (an endosome-dependent process) was examined. As shown in Supplementary Figure S6, hyphal branching were significantly reduced upon Vg treatment.

### 3.5. Effect of endocytosis inhibitors filipin and natamycin on vg-dependent aflatoxin inhibition

**Figure 4:**
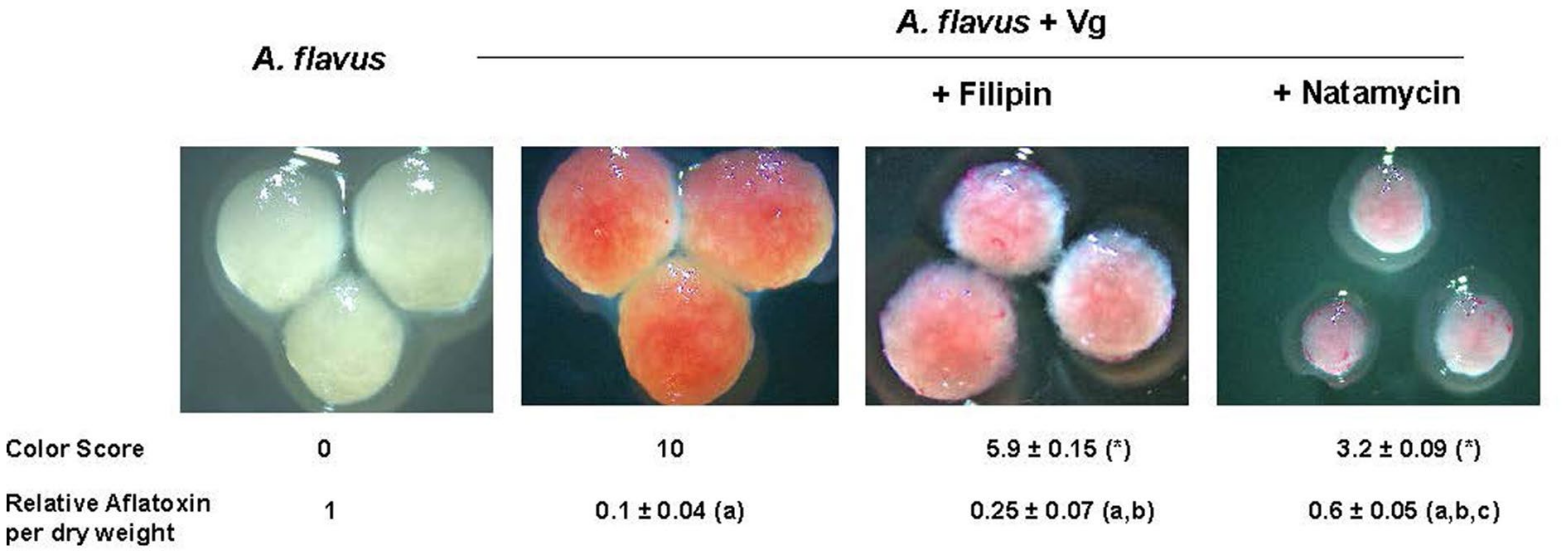
Effect of endocytosis inhibitors filipin and natamycin on uptake of Vg prodigiosin and aflatoxin production. A comparative color scoring technique was used to compare the “redness” of the mycelial pellets that were representative of Vg prodigiosin uptake. Twenty investigators in blinded fashion assigned scored within a scale of 0 to 10, where 0 represented mycelial color of wild-type *A. flavus* and 10 represented color mycelial color upen Vg treatment; *p* < 0.05 Relative aflatoxin per dry weight of mycelia was derived from total aflatoxin levels (ng/g) per gram of mycelia of 40 h time point a, statistically different in comparison with *A. flavus*, b, statistically different in comparison with *A. flavus* +Vg, c, statistically different in comparison with *A. flavus* +Vg (+Filipin). Concentrations of filipin (3μM) and natamycin (6μM) were chosen based on a previous study (Van Leeuwen et al., 2009).

Next, we tested if inhibiting endocytosis uptake of Vg-PG could increase aflatoxin production. Two established endocytosis inhibitors, filipin, and natamycin, were used for this study. As shown in Figure 4, the visual observations corresponded to the color scores. Mycelial color intensity corresponding to Vg-PG uptake dropped in the presence of the inhibitors. The drop in the color score for both inhibitor treatments was statistically significant. Aflatoxin levels correlated well with the levels of color scores. While both inhibitor treatments significantly increased aflatoxin production compared to the untreated control (*A. flavus* + Vg), natamycin appeared to cause a higher increase in aflatoxin levels compared to the filipin-treated samples.

## 4. Discussion

We introduce, through this study, a novel tool for blocking aflatoxin biosynthesis through the disruption of endosomal functions. Our results now provide answers to two critical questions on mechanism of Vg dependent aflatoxin inhibition: (1) whether Vg-prodigiosin is a bioactive component leading to aflatoxin inhibition and (2) whether Vg-prodigiosin uptake by endocytosis leads to aflatoxin inhibition.

To demonstrate that Vg-prodigiosin plays a role in aflatoxin inhibition, we first provided a comparison between prodigiosin producers and non-producers to demonstrate an association between prodigiosin and Vg-dependent aflatoxin inhibition. Next to address the reason of this specificity and determine if prodigiosin was the causal agent responsible for aflatoxin inhibition, we treated the fungus directly with pure prodigiosin and found that prodigiosin could indeed result in significant inhibition of aflatoxin production.

The uptake of Vg-PG into mycelial pellets from the growth medium, was evident from the observation that hyphae turned red in color (which is the color of PG) upon Vg treatment, during which the growth medium loses the reddish color by 40 h of mycelial growth. TEM showed no trace of intact rod-shaped 300–500 nm x 1,000–1,500 nm shaped structures that would be indicative of intact Vg cells inside hyphae, thereby ruling out the possibility of intrahyphal localization intact Vg. We did observe some opaque particulate structures within endosome-like compartments (Supplementary Figure S2) that were not observed in the untreated controls which could be supportive of Vg components (and not intact Vg) reaching inside hyphae and in the endosomes. Under a bright field microscope, Vg-PG could be seen at discrete locations inside the hyphae suggesting the possibility of Vg degradation in the growth medium outside of the fungal cells before Vg-PG internalization; the progressive decrease of OD600 values in the growth medium during Vg treatment supports this possibility (Supplementary Figure S3). Further studies will be undertaken to elucidate the mechanisms leading to Vg degradation during the treatment.

To demonstrate the causal relationship between Vg-PG uptake via endocytosis and aflatoxin inhibition we disrupted Vg-PG endocytosis using two separate endocytosis inhibitors, filipin and natamycin. We show that both filipin and natamycin significantly reduced Vg-PG uptake, and as a result, aflatoxin increased. The different effects of filipin and natamycin on Vg-PG uptake and aflatoxin may be explained by the difference in mechanisms by which the two inhibitors influence *A. flavus* cells. As reported previously, unlike filipin, natamycin inhibits early endocytosis (Van Leeuwen, 2009; Van Leeuwen et al., 2009), possibly resulting in a growth inhibition leading to small pellets and a more drastic inhibition in Vg-PG uptake observed in Figure 4. The observations connected Vg-mediated aflatoxin inhibition directly with endocytosis and suggested that Vg-PG (at least in part) is compartmentalized in endosomes.

Endosomes are critical intracellular compartments that host the enzymes needed for biosynthesis of aflatoxin (Chanda et al., 2009; Hell and Mutegi, 2011; Anindya et al., 2012; Ehrlich et al., 2012; Lim and Keller, 2014; De Ruyck et al., 2015; Keller, 2015; Kistler and Broz, 2015). Hence we investigated if Vg-PG disrupted the normal endosomal functions after reaching endosomes. To test that possibility, we investigated if hyphal fusion was disrupted upon Vg-treatment. Hyphal fusion occurs within and between fungal colonies to form heterokaryons that coexist in a common cytoplasm, and it is currently understood that endosomes trafficking to and from the fusion site assist in the processes that are involved in the fusion (Glass et al., 2004; Riquelme et al., 2007; Upadhyay and Shaw, 2008; Fu et al., 2011; Sanchez-Leon et al., 2015; Fleissner and Herzog, 2016; Daskalov et al., 2017; Zhao et al., 2017; Fischer and Glass, 2019). We observed that Vg treatment prevents hyphal fusion between the uridine and arginine auxotroph mutants TJES19.1 and TJES20.1, respectively, resulting in a significant drop in the formation of heterokaryon containing conidia that could form colonies on GMM. Conidia from fungal colonies obtained from Vg-administered plates looked very similar to conidia obtained from the cross of Δ*hamI* (TXZ9.16) with TJES20.1 strains. An interesting observation connected with the Vg-dependent restriction of hyphal fusion was the significant decrease in hyphal branching within the colonies, which is dependent on the regular functioning of endocytic machinery (Upadhyay and Shaw, 2008; Riquelme et al., 2018). Collectively our data suggested that the fungal cells used existing endocytosis machinery to internalize at least a part of Vg-PG in endosomes, which is associated with the inhibition of hyphal fusion and hyphal branching (both endosome-dependent processes). Since aflatoxin biosynthesis occurs predominantly in endosomal compartments (Chanda et al., 2009; Linz et al., 2012; Lim and Keller, 2014), the observed Vg-dependent aflatoxin inhibition upon Vg-PG localization in endosomes, may be reflective of disruption of endosomal functions that were in line with the significantly reduced mycelial branching.

However, our recent transcriptome studies (Kandel et al., 2022) also show that Vg treatment blocked the expression of all the aflatoxin pathway genes (Kandel et al., 2022) suggesting that Vg-dependent reduction of aflatoxin does not occur only through interference of cellular regulation. The mechanisms by which Vg uptake and endosomal perturbation translate to the downregulation of aflatoxin gene expression and aflatoxin production remain unknown at this time and will be the focus of our future studies. The Vg-induced cell perturbation tool can allow us to study the mechanistic details of how endosomes co-influence secondary metabolism and hyphal development in filamentous fungi.

In summary, the current study unlocks an opportunity for furthering fundamental science and developing prodigiosin-based practical strategies for mitigating aflatoxin and other mycotoxins in food and feed crops. Our promising findings of significant aflatoxin inhibition in Vg-treated corn seeds (Supplementary Figure S2) also provide an initial foundation for our future pilot scale *in- vivo* studies which will test the applicability of the findings in a real-world agricultural setting. We are already in the process of initiating collaborative large-scale studies that will determine the viability of our approach for combating aflatoxin contamination by evaluating critical commercialization factors such as production cost, scalability, environmental impact, and regulatory considerations.

## Data availability statement

The raw data supporting the conclusions of this article will be made available by the authors, without undue reservation.

## Author contributions

AC: conceived the research concept. RJ, JC, ML, RM, PG, TD, SC, and PB: collected data. RJ, JC, ML, RM, PG, AD, NK, and AC: analyzed and interpreted the data. RJ and AC: drafted the manuscript. JC, AD, NK, and AC: revised the manuscript critically for important intellectual content, and all authors provided approval for publication of the final content of the manuscript. All authors contributed to the article and approved the submitted version.

## Conflict of interest

RJ and AC were employed by Mycologics LLC.

The remaining authors declare that the research was conducted in the absence of any commercial or financial relationships that could be construed as a potential conflict of interest.

## Publisher’s note

All claims expressed in this article are solely those of the authors and do not necessarily represent those of their affiliated organizations, or those of the publisher, the editors and the reviewers. Any product that may be evaluated in this article, or claim that may be made by its manufacturer, is not guaranteed or endorsed by the publisher.
