# Supplementary material for: Vibrio gazogenes-dependent disruption of aflatoxin biosynthesis in Aspergillus flavus: the connection with endosomal uptake and hyphal morphogenesis
Source: Front Microbiol. 2023 Sep 8;14:1208961. doi: 10.3389/fmicb.2023.1208961 (PMC10516221; doi:10.3389/fmicb.2023.1208961)
Supplement: Supplementary file 1 [file Image_1.PDF]

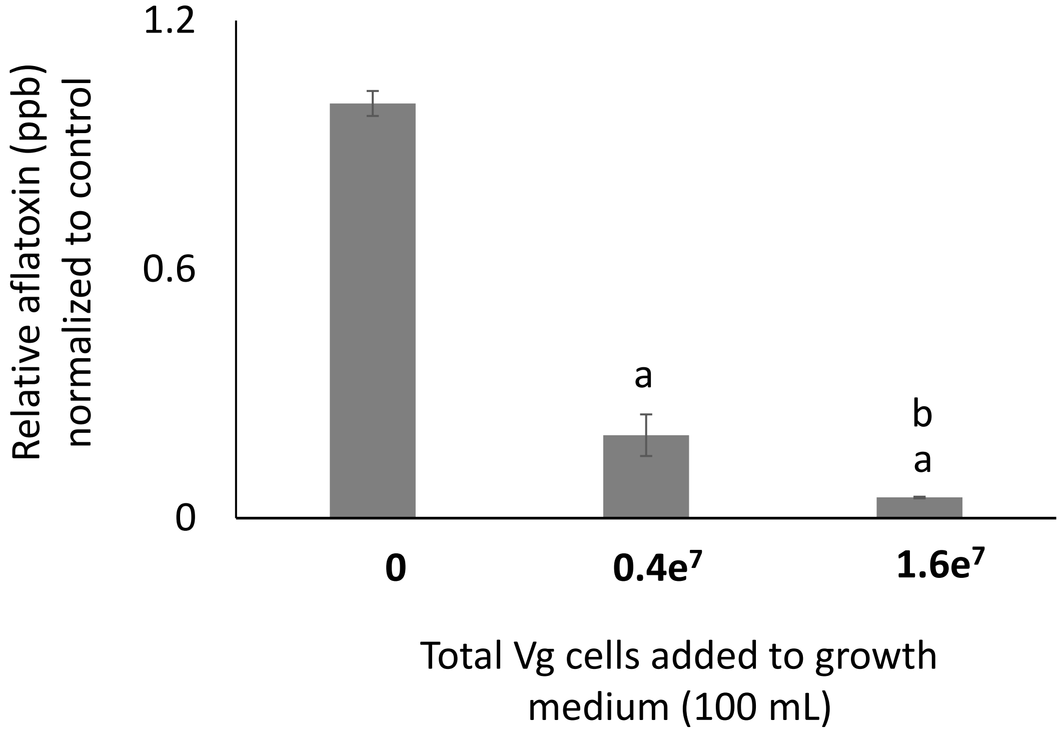


**Figure. S1. Effect of heat-inactivated Vg on aflatoxin production.** Aflatoxin produced per dry weight by *A. flavus* NRRL 3357 in liquid yeast extract sucrose (YES) growth media was compared in the presence and absence of heat-inactivated Vg cells. Two doses, $0.4e^7$ total cells and $1.6e^7$ total cells, were used for the study. Aflatoxin was quantified by ELISA assay after 40 h. The values in the y-axis show the aflatoxin levels relative to the corresponding controls. Error bars, SEM, from triplicate experiments. Statistical significance of two-tailed *p*-values determined using one-way ANOVA for n=3. ($p < 0.05$), a, statistically significant difference compared to untreated (0 cells), b, statistically significant difference compared to dose $0.4e^7$
