# Supplementary material for: Vibrio gazogenes-dependent disruption of aflatoxin biosynthesis in Aspergillus flavus: the connection with endosomal uptake and hyphal morphogenesis
Source: Front Microbiol. 2023 Sep 8;14:1208961. doi: 10.3389/fmicb.2023.1208961 (PMC10516221; doi:10.3389/fmicb.2023.1208961)
Supplement: Supplementary file 2 [file Image_2.PDF]

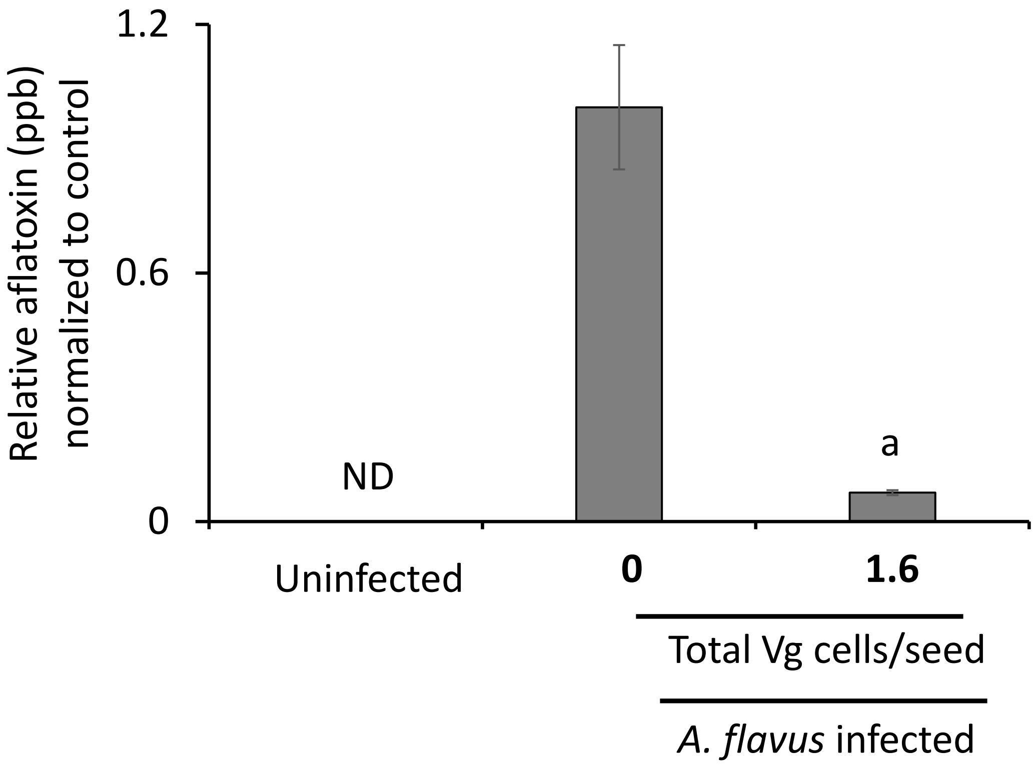

Figure. S2. Effect of Vg on aflatoxin production in *A. flavus* inoculated maize seeds. Comparisons of total aflatoxin produced by *A. flavus* growing on maize seeds (inbred line Va35) in presence (1.6e^{7} total Vg cells/seed) and absence of Vg treatment. The values in the y-axis show the aflatoxin levels relative to the corresponding controls. ND, not detectable, Error bars, SEM, from triplicate experiments. Statistical significance of two-tailed *p*-values determined using an unpaired *t*-test for n=3. ($p < 0.05$). a, statistically significant difference compared to untreated samples (0 cells),
