# Supplementary material for: Vibrio gazogenes-dependent disruption of aflatoxin biosynthesis in Aspergillus flavus: the connection with endosomal uptake and hyphal morphogenesis
Source: Front Microbiol. 2023 Sep 8;14:1208961. doi: 10.3389/fmicb.2023.1208961 (PMC10516221; doi:10.3389/fmicb.2023.1208961)
Supplement: Supplementary file 3 [file Image_3.PDF]

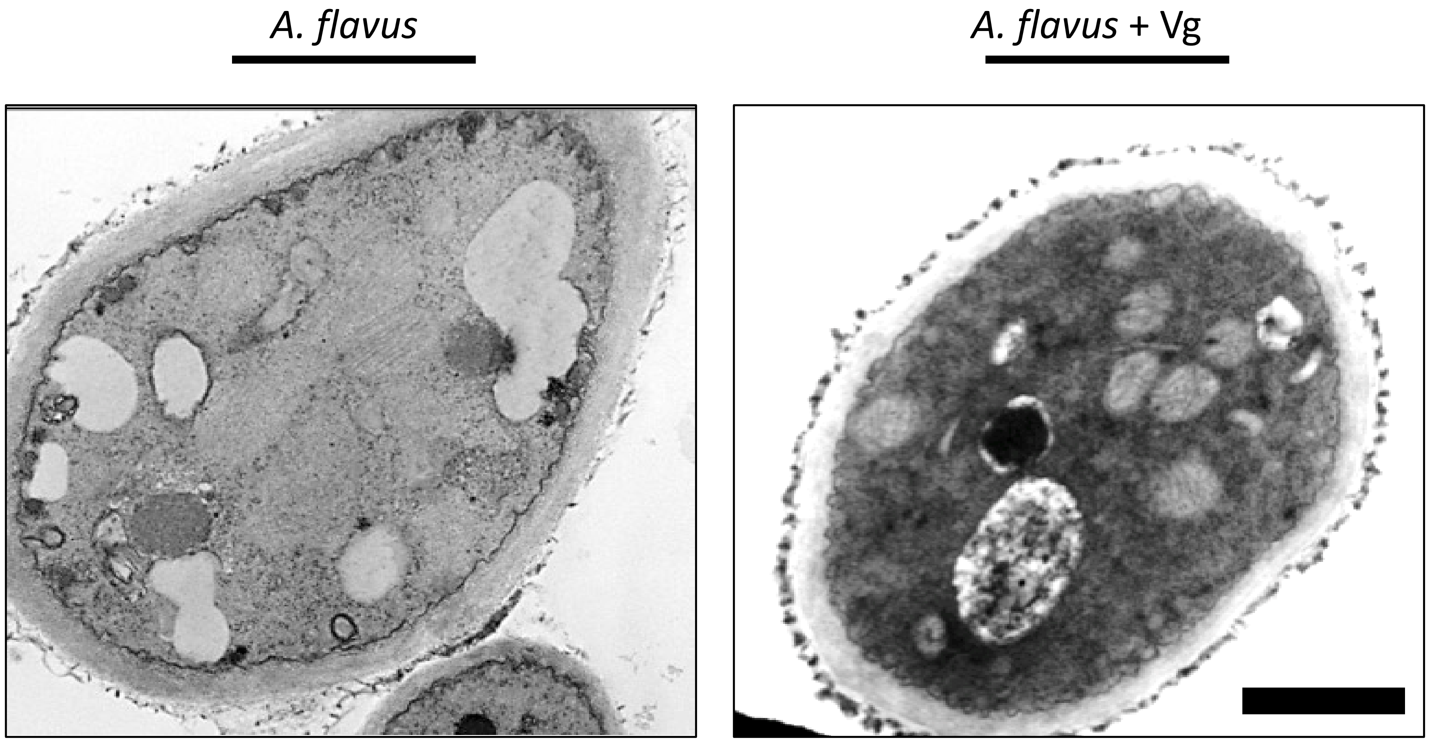

Figure S3. Transmission electron micrographs of *A. flavus*. Representative transmission electron micrographs of 40h *A. flavus* grown in YES liquid medium with and without Vg treatment. Scale bar, 1 μm
