# Supplementary material for: Vibrio gazogenes-dependent disruption of aflatoxin biosynthesis in Aspergillus flavus: the connection with endosomal uptake and hyphal morphogenesis
Source: Front Microbiol. 2023 Sep 8;14:1208961. doi: 10.3389/fmicb.2023.1208961 (PMC10516221; doi:10.3389/fmicb.2023.1208961)
Supplement: Supplementary file 4 [file Image_4.PDF]

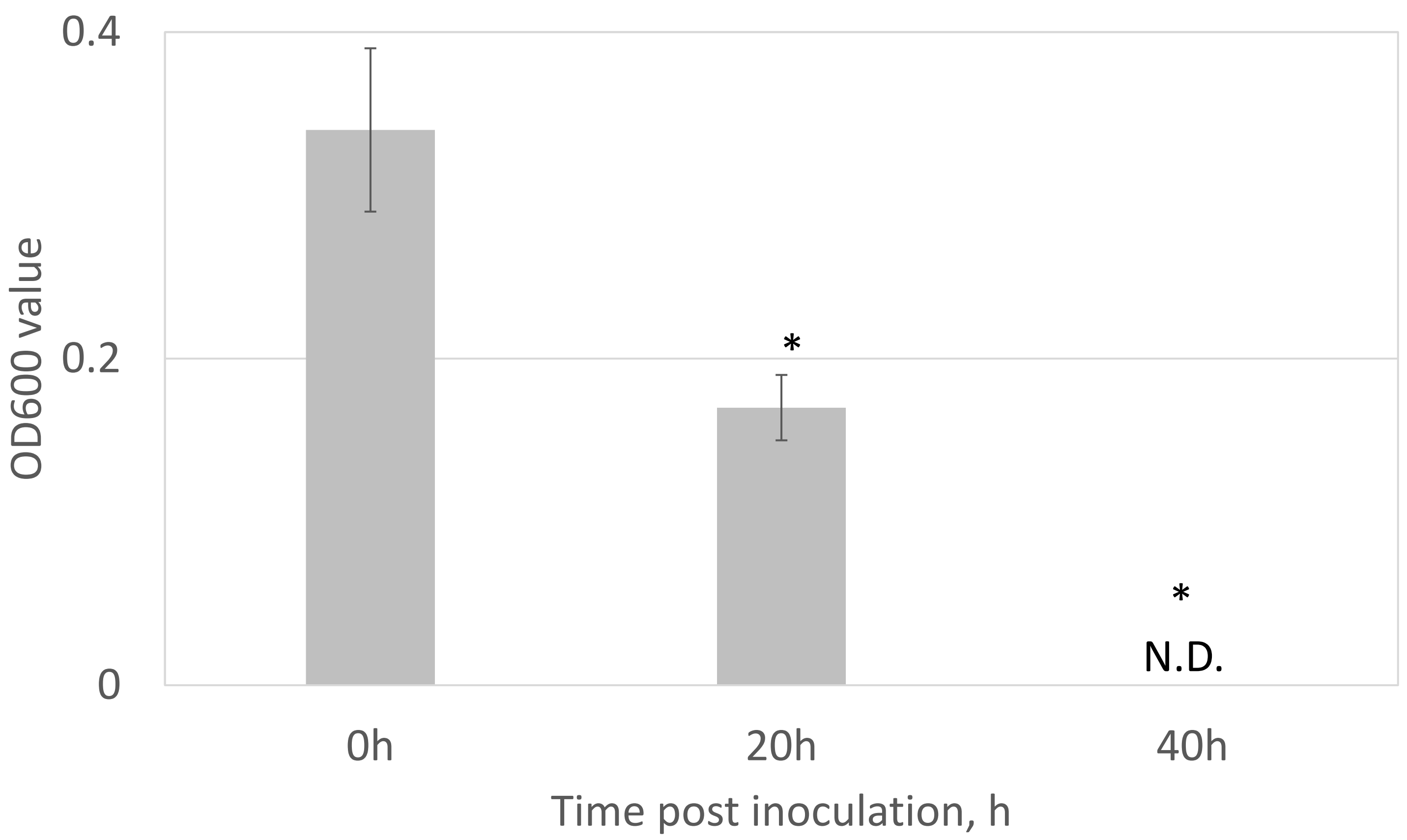

Figure S4. Bacterial concentration measurements of the YES liquid growth medium during Vg treatment. Optical density measurements at 600nm (OD600) of liquid YES growth medium were used to compare Vg concentrations in the growth medium at three different time-points during the Vg treatment. N.D., Not detectable. Error bars represent SEM and the statistical significance of two-tailed *p*-values was determined using one-way ANOVA for n=3. ($p<0.05$), *, statistically significant difference compared to OD600 at the start of treatment.
