# Supplementary material for: Vibrio gazogenes-dependent disruption of aflatoxin biosynthesis in Aspergillus flavus: the connection with endosomal uptake and hyphal morphogenesis
Source: Front Microbiol. 2023 Sep 8;14:1208961. doi: 10.3389/fmicb.2023.1208961 (PMC10516221; doi:10.3389/fmicb.2023.1208961)
Supplement: Supplementary file 5 [file Image_5.PDF]

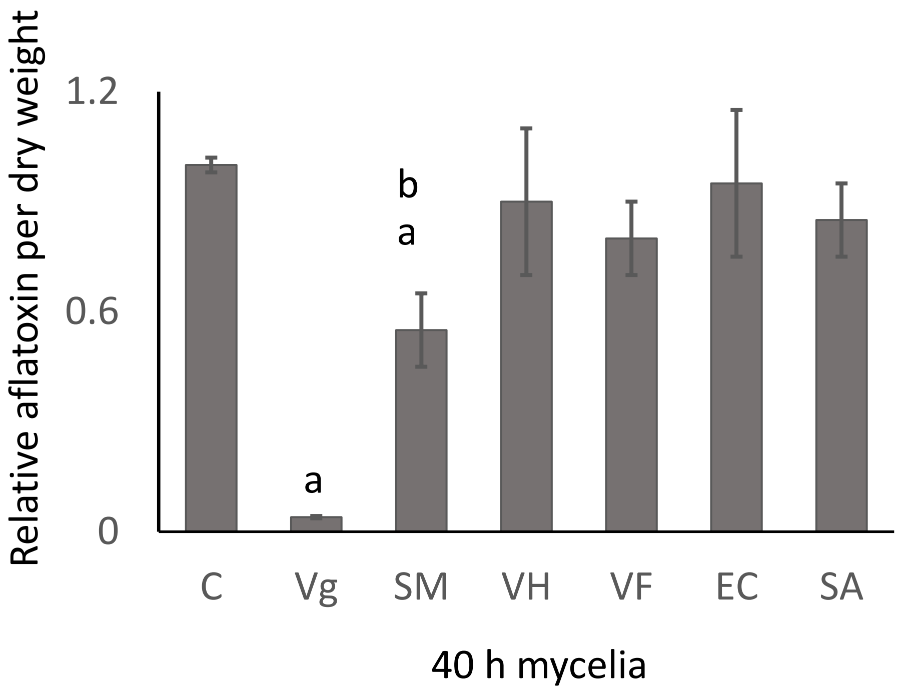

Figure S5. Effect of heat-inactivated cells of prodigiosin producers and non-producers. Aflatoxin produced by *A. flavus* in liquid YES growth media in the presence of heat-inactivated cells of Vg, *Serratia marcescens (SM), two* other Vibrio species, *V. harveyi* (VH), and *V. fischeri* (VF), *Escherichia coli* as a representative Gram-negative bacterium (EC) and *Staphylococcus aureus* as a representative Gram-positive bacterium (SA). Error bars represent SEM from triplicate experiments. Statistical significance of two-tailed *p*-values determined using one-way ANOVA for n=3. (*, $p<0.05$)
