# Supplementary material for: Vibrio gazogenes-dependent disruption of aflatoxin biosynthesis in Aspergillus flavus: the connection with endosomal uptake and hyphal morphogenesis
Source: Front Microbiol. 2023 Sep 8;14:1208961. doi: 10.3389/fmicb.2023.1208961 (PMC10516221; doi:10.3389/fmicb.2023.1208961)
Supplement: Supplementary file 6 [file Image_6.PDF]

	A. flavus	*A. flavus* + Vg
Relative Radial Growth :	1.0	0.3 ± 0.07 (*)
Relative Branching :	1.0	0.6 ± 0.04 (*)

Representative stereomicrographs

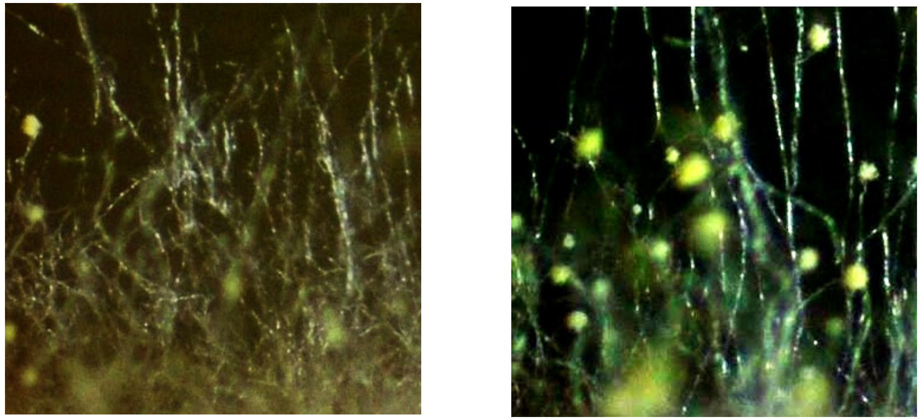

Figure S6. Influence of Vg treatment on radial growth and branching. Comparison of colony morphology with stereo micrographs showing edges of the growing colonies of *A. flavus*, untreated and treated with Vg. Radial growth (measured by an average distance of the tip from the center of the colony), and branching (by visual comparisons) were conducted after 3d of growth. *P<0.05 and considered statistically significant different.
